# Microbiological and molecular studies on a multidrug-resistant *Pseudomonas aeruginosa* from a liver transplant patient with urinary tract infection in Egypt

**DOI:** 10.1186/s12866-024-03318-0

**Published:** 2024-05-27

**Authors:** Mohamed T. Shaaban, Mohamed Abdel-Raouf, Muhammad Zayed, Mahmoud A. Emara

**Affiliations:** 1 Department of Botany and Microbiology, Faculty of Science, Menoufia University, Shebin El- Kom, Egypt; 2 Gastroenterology Surgical Centre, Mansoura University, Mansoura, Egypt

**Keywords:** *Pseudomonas aeruginosa*, Liver transplant, Urinary tract infection, Whole genome sequencing, Antimicrobial resistance, Pangenome

## Abstract

**Background:**

*Pseudomonas aeruginosa* is an opportunistic pathogen responsible for complicated UTIs and exhibits high antibiotic resistance, leading to increased mortality rates, especially in cases of multidrug-resistant strains. This study aimed to investigate the antibiotic susceptibility patterns and genomic characterization of XDR strains identified in end-stage liver disease patients who underwent liver transplants.

**Methods:**

In this study, a number of 30 individuals who underwent liver transplants were registered. Ninety urine and 60 wound site swab samples were collected and processed for culturing, identification, and antimicrobial sensitivity. Extensively drug-resistant strain EMARA01 was confirmed through Sanger sequencing and was then processed for whole genome sequencing to characterize the genomic pattern. Sequencing data were processed for de novo assembly using various tools and databases, including genome annotation, serotype identification, virulence factor genes, and antimicrobial resistance gene. Pangenome analysis of randomly selected 147 reference strains and EMAR01 sequenced strain was performed using the Bacterial Pan Genome Analysis (BPGA) software.

**Results:**

Of these total examined samples, nosocomial infection due to *P. aeruginosa* was detected in twelve patients’ samples. AST analysis showed that *P. aeruginosa* strains exhibit resistance to tobramycin, erythromycin, and gentamicin, followed by piperacillin and ofloxacin, and no strains exhibit resistance to meropenem and imipenem. The CARD database identified 59 AMR genes similar to the EMAR01 strain genome and mostly belong to the family involved in the resistance-nodulation-cell division (RND) antibiotic efflux pump. Five genes; *nalC*, *nalD*, *MexR*, *MexA*, and *MexB*, exhibit resistance to 14 classes of antibiotics, while two AMR; *CpxR*, and *OprM*, exhibit resistance to 15 classes of drugs. Pangenome analysis revealed that the pan-genome remained open, suggesting the potential for acquiring accessory and unique genes. Notably, the genes predominantly involved in amino acid transport metabolism were identified using the KEGG database.

**Conclusions:**

This study provides valuable insights into the antimicrobial resistance profile, genetic features, and genomic evolution of *P. aeruginosa* strains causing UTIs in liver transplant patients. The findings emphasize the significance of comprehending AMR mechanisms and genetic diversity in *P. aeruginosa* for developing effective treatment strategies and infection control measures.

**Supplementary Information:**

The online version contains supplementary material available at 10.1186/s12866-024-03318-0.

## Background

Liver transplantation surgery is a life-extending procedure for individuals suffering from end-stage liver disorder (ESLD). Opportunistic microbial infections are common during the initial year following liver transplantation surgery, primarily because the immune systems of individuals are suppressed by regular medications such as corticosteroids, which aid in the acceptance of the transplanted organ [1–3]. Among Liver transplant patients, bacterial infections reign as the predominant type, accounting for 70% of all infections observed, followed by fungal and viral infections. The risk of infection fluctuates depending on the postoperative timeframe, this temporal aspect plays a significant role in determining the susceptibility to the microbial infections [4]. Urinary tract infections (UTIs), bacteraemia, and pneumonia stand out as the most prevalent infections in the postoperative patients [5].

*Pseudomonas aeruginosa* is a formidable opportunistic human pathogen that can cause severe acute and chronic infections, particularly in immune-compromised patients, and is also responsible for the leading cause of complicated urinary tract infections (UTIs) [6, 7]. Such complicated UTIs due to *P. aeruginosa* strains often follow a progressive course because of their adaptability to various stress conditions and multifactorial resistance [8]. The high intrinsic antibiotic resistance mechanism of *P. aeruginosa* and its flexibility to develop new resistance during antibiotic treatment enables it to overcome many antibiotics [9]. Non-resistant strains are responsible for high mortality rates of up to 23% in UTI patients, while the mortality rate increases substantially to approximately 67% in cases of infection caused by multi-drug resistant (MDR) strains [10]. *P. aeruginosa* can harbour and accrue numerous resistance determinants resulting in frequent MDR [11]. *P. aeruginosa* isolated from organ transplant recipients exhibits significantly elevated rates of carbapenem resistance compared to those isolated from non-transplant recipients [12]. This finding highlights the tendency of *P. aeruginosa* to develop resistance mechanisms in individuals who have undergone organ transplantation. Reports in liver transplant recipients are often in the setting of single centre outbreaks or high local prevalence [13]. Rates of *P. aeruginosa* bloodstream infections in liver transplant range from 6.5 to 10%, with over 50% of isolates demonstrating multiple drug resistance [14]. Although recommended by many experts, routine combination therapy’s role in treating *P. aeruginosa* infections remains controversial [15].

Recently, the emergence of MDR and extensively drug-resistant (XDR) bacterial strains as a result of the evolution of many resistance genes such as lactamases, 16 S rRNA methylases, and carbapenems has resulted in incurable microbial infections with serious global threats to human health, underscoring the need for novel treatment approaches. The most prevalent form of *P. aeruginosa*-acquired resistance is the development of carbapenems, which impart resistance to most commercially available B-lactams [16]. The main defence mechanisms employed by *P. aeruginosa* against antibiotic assault may generally be divided into intrinsic, acquired, and adaptive resistance [17]. The low permeability of the outer membrane, the development of efflux pumps that expel drugs from the cell, and the creation of antibiotic-inactivating enzymes are all characteristics of *P. aeruginosa* intrinsic resistance [18].

The advancements in DNA sequencing technology has enabled the utilization of whole genome sequencing (WGS) in identifying, examining the evolutionary patterns of *P. aeruginosa* and investigating its epidemiology within healthcare facilities [19]. WGS has emerged as a cutting-edge approach to studying resistance mechanisms in bacteria, providing comprehensive insights into the genetic makeup of the pathogens. Moreover, this technique enables the simultaneous processing of vast amounts of DNA sequences in a high-throughput manner, reducing both time and resource requirements [20].

Herby, we systematically screened the presence of *P. aeruginosa* in both urine and stool specimens obtained from the liver transplant patients, both prior to and following the surgery. Additionally, we identified the antimicrobial resistance (AMR) genes in Liver transplant patient with UTI by sequencing the whole genome of the *P. aeruginosa* EMARA01 strain that exhibits resistance to various antibiotics from the liver transplant patient with urinary tract infection. This strain demonstrated heightened resistance to a broader spectrum of antibiotics compared to other *P. aeruginosa* isolates in this study. Genome sequencing analysis revealed 59 AMR genes involved in resistance against various drug classes, and with various resistance mechanisms. Pangenome analysis of EMARA01 with 147 randomly selected reference *P. aeruginosa* genomes of clinical isolates was performed to analyse both its genomic evolution and genetic diversity. To the best of our knowledge, this is the first study for the comparative genome analysis of *P. aeruginosa* strains obtained from liver transplant patient in Egypt, and as compared with other Arabian countries, and diverse geographical regions.

## Materials and methods

### Sample Collection and ethical approval

A cohort of 30 end-stage liver disease patients who had undergone liver transplants between March 2021 to December 2021 at the Gastroenterology Surgical Center (GEC), Mansoura University Hospital, Egypt, was included in the current study. The study was conducted according to the principles of the Declaration of Helsinki. Approval code MDP.23.08.129 was acquired from the Ethical Committee from the Faculty of Medicine, Mansoura University, Egypt. Following the acquisition of written informed consent from all of the participants in this study, urine samples were collected from each registered patient according to the specified criteria: one week before the liver transplant, one week after the transplant, and two weeks post-transplant, while swab samples at the surgical wound sites were obtained after one week and two weeks of postoperation. All samples were transported to the clinical microbiology laboratory within two hours to ensure timely analysis. Samples were analyzed for the identification of nosocomial infection due to *P. aeruginosa.*

### Bacterial isolation, identification, and Antibiotic Susceptibility Test (AST)

All samples were initially processed for Gram staining and followed by culturing. The Gram-positive bacteria were inoculated using differential growth medium Blood Agar [21], while the Gram-negative bacteria were cultured in the cystine lactose electrolyte deficient agar (CLED) and MacConkey agar [22, 23] The inoculated plates were incubated aerobically at 37 °C for 24 h, and the isolated colonies were evaluated for colony morphology and then were examined for identification based on biochemical and molecular evaluation. For biochemical identification, the isolated colonies were examined for catalase [24], oxidase [25], triple sugar iron [26], indole [27], H_2_S production, and motility. Furthermore, it was further confirmed using an analytical profile index (API) [28]. Additionally, the bacterial DNA was isolated for molecular identification using DNeasy® Blood & Tissue Kit [29, 30]. The quality of extracted DNA was evaluated by Agarose gel electrophoresis [31], and the concentration was estimated by nanodrop. The targeted region (16 S DNA) was amplified by using two sets of universal primers; [785-For Primer GGATTAGATACCCTGGTA; & 907-Rev Primer: CCGTCAATTCMTTTRAGTTT] and [27-For Primer: AGAGTTTGATCMTGGCTCAG & 1492-Rev Primer: TACGGYTACCTTGTTACGACTT] [32, 33] The amplified products were confirmed by 2% Agarose gel electrophoresis. Amplified samples with intact bands were processed using GeneJET™ PCR purification kit (Thermo Scientific, USA) followed by chain termination reaction/Sanger sequencing using BigDye Terminator v.3.1 cycle sequencing kit as per manual instruction. Both strands were examined for chain termination reaction/Sanger sequencing to obtain the consensus sequences. The sequencing product was purified by ethanol precipitation and sequencing using an ABI Genetic analyser. The obtained consensus sequences and the sequencing data were analysed using BioEdit v 7.0 software [18]. The consensus sequences were processed for BLAST analysis to confirm the species with maximum resemblance.

The isolated colonies from the different culture media were examined for an antimicrobial sensitivity test (AST) using the Kirby Bauer technique [34] as per the guidelines of the Clinical and Laboratory Standards Institute (CLSI) [35]. For AST analysis, we examined the following antibiotics, including ciprofloxacin (CIP 5 µg), norfloxacin (NOR 10 µg), ofloxacin (OFX 5 µg), imipenem (IPM 10 µg), meropenem (MEM 10 µg), piperacillin (PRL 100 µg), ceftazidime (CAZ 10 µg), gentamicin (CN 10 µg), amikacin (AK 30 µg), tobramycin (TOB 10 µg) and erythromycin (E 15 µg) were examined for AST. Finally, the genomic characterization was performed for the most antibiotic-resistant *P. aeruginosa* EMARA01 strain, isolated from the patient’s urine sample.

### Whole Genome Sequencing and Annotation

Whole genome sequencing of MDR *P. aeruginosa* EMARA01 strain was performed using 2*151 bp flow cell chemistry on the NovaSeq Illumina. The quality of short-sequenced raw reads was evaluated by the FASQC tool [36]. Adapter sequences and the poor-quality data were trimmed using Trimmomatic [37] software with default parameter settings. The quality cut-off value was Phred33. *P. aeruginosa* genome assembly was performed using BWA MEM package [38] and the percentage of the covered draft genome was determined by samtools [39]. The quality of the EMARA01 strain sequenced data aligned against the PA01 reference genome strain was evaluated by Qualimap v 2.3 [40].

The sequenced data was processed for *de novo* assembly using Unicycler [41], and the accuracy of assembled genome was improved by Pilon [42]. The quality of the assembled draft genome was assessed by QUAST [43]. The annotation of the sequenced EMARA01 strain was performed by NCBI Prokaryotic Genome Annotation Pipeline (PGAP), as well as PATRIC v 3.6.12, and rapid annotation using Subsystem Technology 2.0 (RAST) online tools [44]. The genomic visualization of the bacterial genome was performed by the Proksee tool [44].

The *Pseudomonas aeruginosa serotyper (PAst)* 1.0 tool (https://cge.food.dtu.dk/services/PAst) was employed for serotyping and serogrouping analysis, while the PathogenFinder tool [45] was used to determine the strain’s pathogenicity. To predict the antimicrobial resistance (AMR) genes in the EMARA01 strain, an in-silico method was employed using various databases, including the Comprehensive Antibiotic Resistance Database (CARD) [46], ResFinder 4.1 [47] (https://cge.cbs.dtu.dk/services/ResFinder). The BacAnt (http://bacant.net/BacAnt/) was also searched to identify resistance genes, introns, and transposable elements with 90% identity and 60% coverage [48]. The presence of plasmids was investigated using the plasmid database (PLSDB) [49], and virulence genes were identified using the virulence factor database (http://www.mgc.ac.cn/VFs). The antiSMASH v7.0.0 tool was utilized for secondary metabolites identification [50], and BAGEL4 was used to search genes coding bacteriocin [51].

### Pangenome Analysis

The pan-genome and core genome statistics of the 148 *P. aeruginosa* strains were assessed using BPGA [52]. This analysis aimed to identify strain-specific genomic features and to analyse the genomic diversity among the selected strains. The study utilized genome sequences of 147 *P. aeruginosa* strains obtained from 30 countries belonging to various geographic regions, mostly from Egypt, Lebanon, India, China, Saudi Arabia, USA, Switzerland, and Turkey, sourced from the NCBI database along with one in-house sequenced strain (EMARA01).

Gene clustering into families was performed by the USEARCH clustering algorithm, and the sequence identity was selected as 70%. The pan-core plot was constructed with 30 combinations, and the coding sequences were aligned by MUSCLE. Core and pangenome phylogeny were plotted by the Neighbour-Joining method, and the phylogenetic visualization was done using web- based iTOL (https://itol.embl.de/).

### Biological function and Pathways Identification

The BPGA’s pan-genome functional analysis module was utilized to assign cluster of Orthologous Genes (COG) and Kyoto Encyclopaedia of Genes and Genomes (KEGG) classifications to the core, accessory, and unique gene families. The KEGG is a collection of databases dealing with genomes, gene function, biological pathways, diseases, drugs, and metabolism. Genes involved in the function pathways were also identified using the RASTtk annotation tool.

## Results and discussion

### Bacterial isolation, Identification and AST

The consent of thirty liver transplant patients in this study was taken. In this study, urine specimens were collected from patients at various time points before and after liver transplant surgery, along with wound site samples obtained at the corresponding intervals post-transplantation. Following analysis, *P. aeruginosa* was identified in a subset of patient samples, as it was detected in four urine samples and eight wound site samples collected from one week of the surgery. Gram staining result showed Gram-negative rods. The isolated bacterial colonies were tested positive for oxidase, citrate and negative for urease, indole, and H_2_S. Further, the colonies were identified as *P. aeruginosa* based on API. API results indicate that the EMARA01 strain showed a positive reaction with ADH (decarboxylation of the amino acid arginine by arginine dihydrolase), citrate, hydrolysing the gelatine (indicating the presence of gelatinase), and nitrate reduction (Supplementary Fig. 1).

Of the twelve *P. aeruginosa* strains, four isolated from urine samples and eight identified from wound site swab samples were tested for drug sensitivity and resistance. The tested result indicated that all twelve strains exhibit resistance to tobramycin, erythromycin, and gentamicin, followed by piperacillin, and ofloxacin, and no strains exhibit resistance to meropenem and imipenem. Despite the historical prominence of aminoglycoside antibiotics such as tobramycin and gentamicin in the treatment of *P. aeruginosa* infections, it is surprising that all isolates in this study were resistant to these drugs. It is conceivable that prolonged historical usage of these antibiotics has facilitated the development of resistance in *P. aeruginosa*. This resistance may arise through diverse mechanisms, such as the production of enzymes that deactivate the antibiotics or through efflux pumps. Therefore, the exploration of combination therapy or the pursuit of novel therapeutic approaches is strongly encouraged to address this challenge. The details of all tested 11 antibiotics tested among all *P. aeruginosa* strains isolated from urine samples and wound site swab isolated from liver transplant patient is shown in (Table 1).

**Table 1:** Antibiotic sensitivity test by using disk diffusion method according to CLSI 2018

Name of Antibiotics	µg	Resistance (mm)	Intermediate (mm)	Susceptible (mm)	Zones of Inhibition (mm)												
					Isolated from urine samples				Isolated from wound site swab samples								
(OXOID)																	
					EMARA01	PU02	PU03	PU04	PW05	PW06	PW07	PW08	PW09	PW 10	PW 11	PW 12	PW 13
Ciprofloxacin (CIP)	5	≤ 15	16–20	≥ 21	R	I	I	S	S	S	I	S	S	R	S	S	S
Norfloxacin (NOR)	10	≤ 12	13–16	≥ 17	R	R	I	S	S	I	I	S	S	I	S	I	S
Ofloxacin (OFX)	5	≤ 14	15–21	≥ 22	R	I	R	I	I	I	R	S	I	R	I	R	I
Meropenem (MEM)	10	≤ 13	14–15	≥ 16	S	S	S	S	S	S	S	S	S	S	S	S	S
Imipenem (IPM)	10	≤ 13	14–15	≥ 16	S	S	S	S	S	S	S	S	S	S	S	S	S
Ceftazidime (CAZ)	10	≤ 14	15–17	≥ 18	R	R	R	R	R	R	R	S	R	R	R	S	S
Amikacin (AK)	30	≤ 14	15–16	≥ 17	S	S	S	S	S	S	I	S	S	S	S	S	S
Tobramycin (TOB)	10	≤ 12	13–14	≥ 15	R	R	R	R	R	R	R	R	R	R	R	R	R
Erythromycin (ERY)	15	≤ 13	14–17	≥ 18	R	R	R	R	R	R	R	R	R	R	R	R	R
Gentamicin (CN)	10	≤ 12	13–14	≥ 15	R	R	R	R	R	R	R	R	R	R	R	R	R
Piperacillin (PPL)	100	≤ 14	15–17	≥ 18	R	R	R	S	S	R	R	R	R	R	S	R	S

R = Resistance, I = Intermediated, S = Susceptible

*P. aeruginosa* is recognized as a prominent contributor to nosocomial infections. A comprehensive study of isolates’ susceptibility to antimicrobial drugs is necessary to reduce the spread of this antimicrobial-resistant microorganism and to implement effective infection control strategies [53]. The analysis of antimicrobial susceptibility testing (AST) in the study disclosed that all strains were responsive to meropenem and imipenem, yet resistant to tobramycin, erythromycin, and gentamicin antibiotics. The four scrutinized antibiotics including amikacin, ciprofloxacin, meropenem, and imipenem, were identified as crucial components of anti-pseudomonal agents, exhibiting diverse levels of susceptibility among the isolates. All isolates were susceptible to meropenem, and imipenem, 92% were sensitive to amikacin, and 66% showed susceptibility to ciprofloxacin. Exploring the fluoroquinolone family, ofloxacin revealed a spectrum of responses: one isolate was susceptible, seven displayed intermediate resistance, and five exhibited full resistance among the twelve isolates. Turning attention to the quinolone family, norfloxacin demonstrated six isolates with susceptibility, five with intermediate resistance, and two with resistance (Table 1). In this study, more than 70% of the *P. aeruginosa* isolates exhibited resistance to ceftazidime (77%), piperacillin (75%), tobramycin (100.0%), and gentamicin (100.0%). These results were in line with previous reports from Iran [54], Egypt [55], USA [56], Kingdom of Saudi Arabia [57, 58], Malaysia [59]. It is noted that there was low resistance rates for ceftazidime was noted in Iran [60]. The variation could be attributed to distinctions in population characteristics, including type and race, as well as variations in isolate sources.

The phenotypic characterization of the antimicrobial resistance patterns of the *P. aeruginosa* EMARA01 identified in the urine sample collected before one week of the liver transplant exhibit resistance to numerous clinically available important antibiotics except for amikacin and two β-lactam antibiotics; meropenem and imipenem. This MDR EMARA01 strain was confirmed at the molecular level through 16 S rRNA (Supplementary Fig. 2). The gel image confirmed the amplified products, which were then processed for Sanger sequencing using both targeted regions. The consensus sequences were processed for BLAST analysis, and it is noted that the EMARA01 strain exhibits high nucleotide similarity to *P. aeruginosa* species. The sequenced data with read length 1356 nucleotides of 16 S rRNA gene (accession no. OQ976904) exhibit the closest similarity to *P. aeruginosa* various strains such as WS02, UASR_4, P2, SJC-03, and P4. Hence, the confirmed MDR *P. aeruginosa* EMARA01 was selected for complete genome sequencing.

### Genome annotation of *P. Aeruginosa* EMARA01

The paired-end sequencing data obtained from the sequencing platform comprised 18,470,938 short reads in fastq format. After FASTQC analysis, all reads underwent pre-processing using the trimmomatic package with default parameters. This process involved removing adapter sequences, low-quality regions (especially towards the ends of the reads), and bases with a Phred score of less than 33. Out of the total filtered reads, 17,627,341 short-sequenced reads were aligned against the reference strain PA01 comprised of 6,264,404 nucleotide bases. Of the total mapped reads, 8,841,139 were first in pair and 8,786,202 s in pair, while 107,081 reads singleton in pair. The mean mapping quality of the EMARA01 strain against the reference genome was 59.37 and the sequence read depth was 411.2. The coverage of each base against the reference genome PAO1 strain is shown in (Supplementary Fig. 3).

**Fig. 1:**
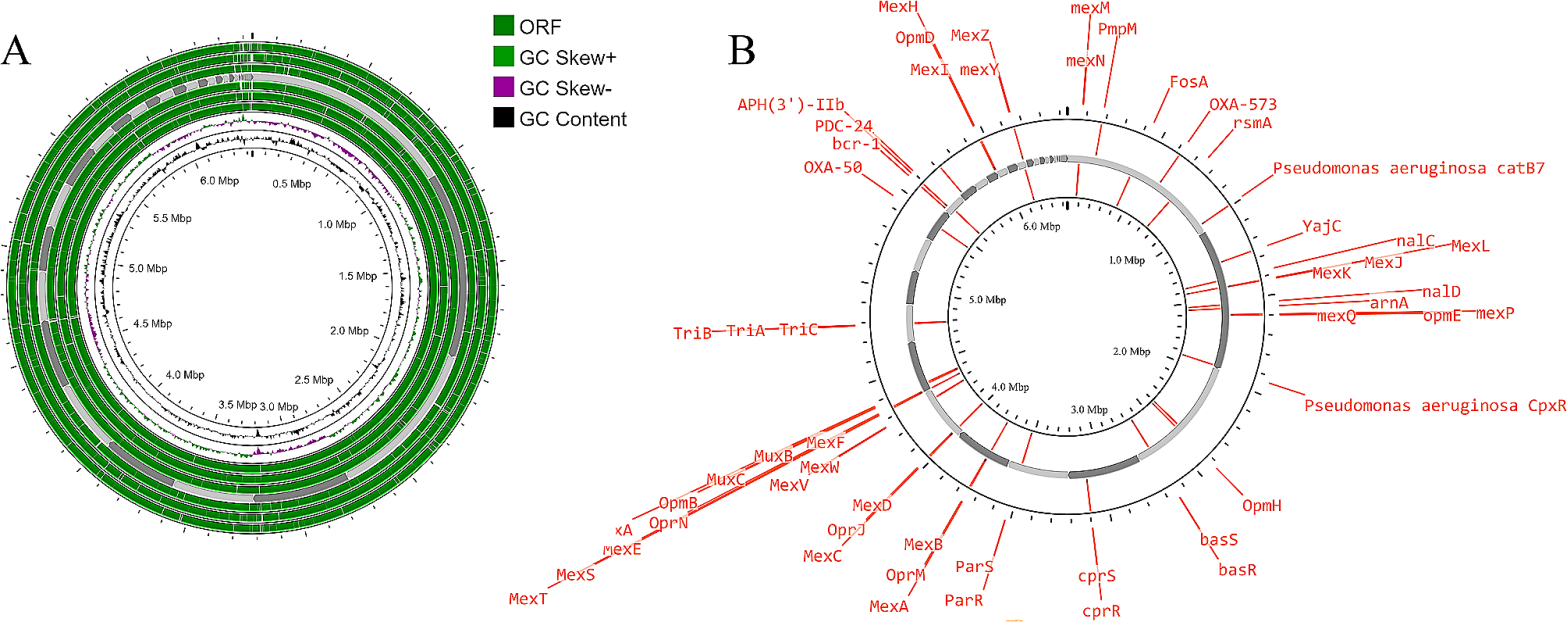
Circular genomic organization and characterization of *P. aeruginosa* EMARA01 strain. In **A** first three circle exhibit CDSs in forward strand, a contigs layer, three CDSs in reverse strand, two layers of GC skew and last circle representing GC content. **B** representing the position of AMR identified by using CARD database

The filter-sequenced reads were further processed for *de novo* assembly using Unicycler. The quality of the assembly was improved by Pilon, resulting in a final assembly of the *P. aeruginosa* EMARA01 genome with 66 contigs, each having a length greater than 300 bases. The assembled genome of *P. aeruginosa* EMARA01 (accession No. JARQZF000000000.1) was composed of 6,438,302 nucleotides distributed among the 66 contigs, with a GC content of 66.38%. The largest contig had a length of 1,044,180 bases, while the contig with the N50 value had a length of 394,570 nucleotides. The contig L50 value was 5, indicating the number of contigs needed to reach half of the genome length. Functional annotation of EMARA01 using PGAP identified 5,928 coding sequences (CDSs) and 65 RNA sequences. Among the 5,928 CDSs, 5,893 encoded proteins, while 35 were pseudogenes that did not code for any proteins. Of the 65 total RNA sequences, 58 were tRNA, and three were rRNA (two complete rRNA, including one 16 S, one 23 S, and one partial 5 S rRNA). The protein features included 5,416 previously characterized proteins and 477 hypothetical proteins. The non-ribosomal peptide synthase/polyketide synthase had the highest number of amino acids (*n* = 4,989), while the pyrroloquinoline quinone precursor peptide PqqA consisted of only 23 amino acids in the sequenced genome. The circular representation of the assembled *P. aeruginosa* EMARA01 genome visualized by Proksee is shown in (Fig. 1).

PathogenFinder analysis showed that the sequenced EMARA01 strain predicted as pathogenic with a probability of being a human pathogen was 0.75 out of 1. The *P. aeruginosa* serotyper (PAst) is utilized for in silico serotyping of *P. aeruginosa* isolates into 1 of 11 serogroups (covering the 20 serotypes). The PAst is highly efficient in silico serotyping tool that predicts serogroup based on the sequence of the O-specific antigen (OSA) gene cluster. Here, PAst v1.0 identified that the EMARA01 strain belongs to the O7 serogroup.

### Antimicrobial Resistance Profiling

The functional annotation of the EMARA01 strain using the CARD databases revealed the presence of numerous genes that confer resistance to various antibiotics. Based on the CARD database analysis, 59 AMR genes were identified by resistance gene identifier, only selecting perfect and strict criteria (Fig. 1B). Due to its poor outer membrane permeability and aggressive antibiotic efflux, *P. aeruginosa* is renowned for its inherent resistance to numerous front-line medicines [61–63]. Out of 59 AMR genes, 44 genes belong to a family that is involved in resistance-nodulation-cell division (RND) antibiotic efflux pump, five genes; *basS, cprR, cprS, arnA*, and *basR*, belong to *pmr* phosphoethanolamine transferase gene family while the remaining ten belongs to each a single AMR gene family. Based on the protein homology model, four AMR genes, including *nalC, nalD, MexR, MexA*, and *MexB* exhibit resistance to 14 class of antibiotics namely macrolide antibiotic, fluoroquinolone antibiotic, monobactam, carbapenem, cephalosporin, cephamycin, tetracycline antibiotic, peptide antibiotic, aminocoumarin antibiotic, diaminopyrimidine antibiotic, sulfonamide antibiotic, phenicol antibiotic, penems. Two AMR genes, *Pseudomonas aeruginosa CpxR*, and *OprM*, resist these antibiotics besides cephamycin.

AMR gene profile suggests that the presence of resistome (ARGs) and associated resistance mechanisms in *P. aeruginosa* EMARA01, along with beta-lactam genes and efflux pump systems, may contribute to this bacterium’s extensive resistance to nearly all antibiotics used for treatment purposes and may even be the reason for its emergence as a pan drug-resistant bacterium in Egypt. Due to its numerous intrinsic defence mechanisms against antibiotics, *P. aeruginosa* EMARA01 is a particularly dangerous bacterium. According to a preliminary analysis of the distribution of a few genes involved in antibiotic resistance (*oprM, ampC, ampD*, and *PIB-1*) and functionally annotated using the *Pseudomonas* Genome Database, these genetic entities are identified in the in-house sequenced *P. aeruginosa* EMARA01 strain.

These findings are consistent with those of the *P. aeruginosa* Pa1242 strain, where it was shown that all four b-lactamase classes coexisted, were inherited chromosomally, and acquired a variety of resistance determinants, are the leading efflux pump systems conferring resistance to *P. aeruginosa* EMARA01. The resistance of this bacteria can be further increased by its ease of acquiring genetically encoded resistance determinants from other diseases. Through these efflux pump systems, clinically important *P. aeruginosa* strains demonstrate resistance to various medicines, including fluoroquinolone, rifamycin, cephalosporin, glycylcycline, tetracycline and glycopeptide antibiotic. *MexAB-OprM, MexCD-OprJ, MexEFOprN*, and *MexXY/OprM* are examples of tripartite efflux systems that are among the inherent and acquired resistance mechanisms [61] causing the extrusion of xenobiotics and antimicrobials from the inside of the cell. *MexA* contributes significantly to *P. aeruginosa* inherent resistance to numerous antibiotics including macrolide, fluoroquinolone, monobactam, carbapenem, cephalosporin, cephamycin, tetracycline, diaminopyrimidine antibiotic, sulfonamide antibiotic, phenicol antibiotic other structurally unrelated antimicrobial compounds, and various structurally unrelated antibacterial substances (Supplementary Table 1). *MexEF-OprN* can extrude quinolones [64], and *MexXY-OprM* may eject aminoglycosides and cephalosporins from inside bacterial cells [65]. However, no plasmid sequence was identified using PLSDB in the EMARA01 strain.

Genome annotation using antiSMASH v7.0, used to detect and characterize biosynthetic clusters, predicted the gene clusters, including especially NRP, and Polyketide for several antimicrobial compounds, including azetidomonamide A/azetidomonamide, pyoluteorin, pseudopaline, L-2-amino-4-methoxy-trans-3-butenoic acid, pyochelin, and others. BEAGAL analysis identified two areas of interest; 15.9.AOI_01 for class Bottromycin and 1.15.AOI_01 for class 91.3; Pyocin-S1. Based on nucleotide similarity, BacAnt analysis of EMARA01 strains identified 21 AMR, 15 transposons, and two integrons.

### Pan and Core Genome statistics

The versatility of the studied strains is evaluated by assessing the open or closed nature of the pan-genome. The expected gene number in both the pan-genome and core-genome was calculated using curve fitting based on Heaps’ law. The equation Ypan = Apan * x^Bpan + Cpan represents the pan-genome, where Y is the pan-genome size, x is the number of genomes, and Apan, Bpan, and Cpan are fitting parameters. Similarly, the core-genome is represented by the exponential equation Ycore = Acore * e^(-Bcore * x) + Ccore. In this equation, Bpan determines whether the pan-genome is closed (Bpan < 0 or Bpan > 1) or open (0 < Bpan < 1).

A total of 148 strains were analysed, and the genomic data of 81 strains with complete genomes and 67 strains with contigs and scaffolds were obtained from NCBI. The genome size of all strains was observed to be 6.85 ± 0.289 MB, with a GC content of 66.024 ± 0.27. Additionally, the average protein sequences consisted of 6205.4 ± 289.385.

**Fig. 2:**
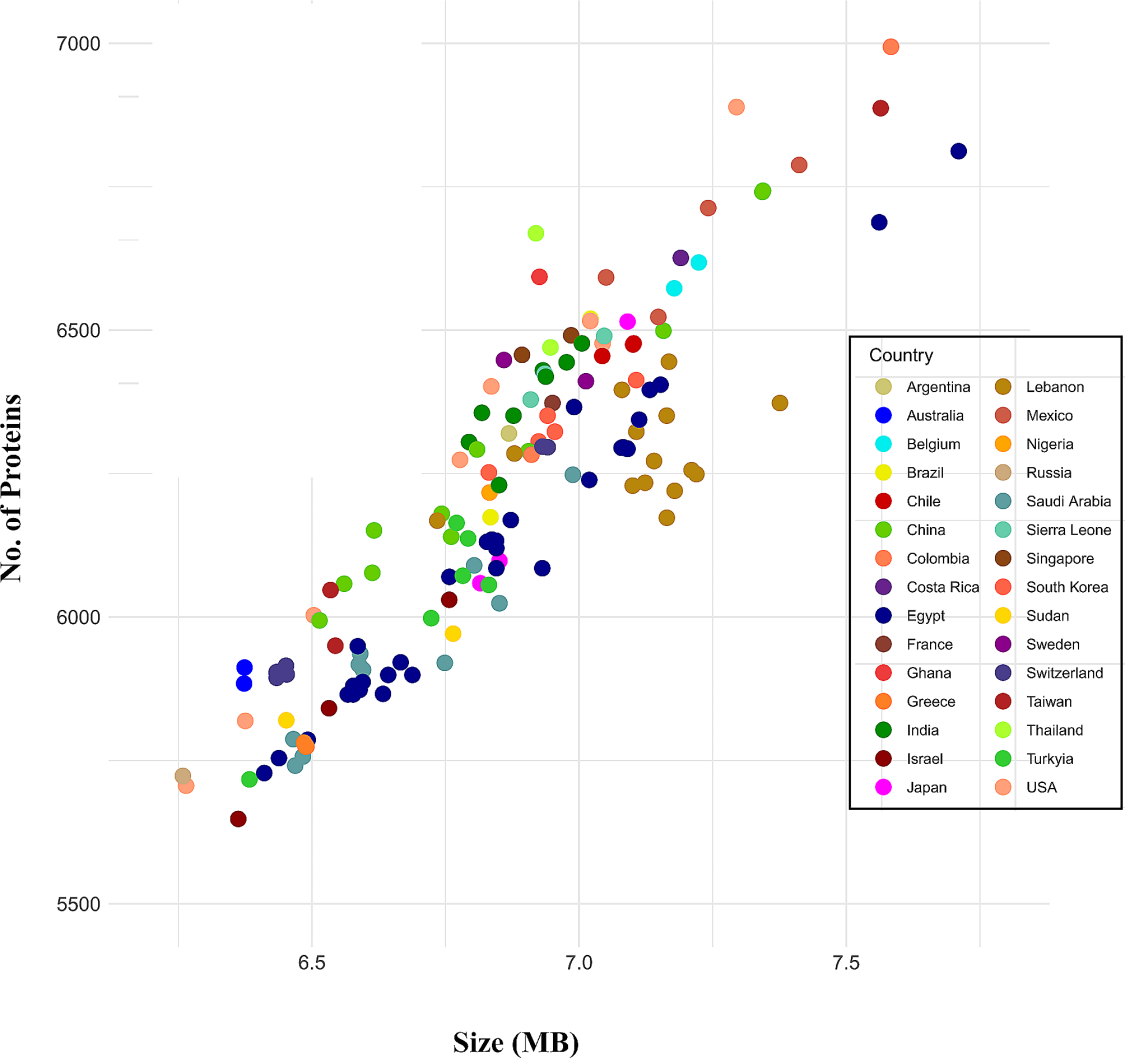
Distribution of coding sequences in the studied strains with respect to the genome size

**Fig. 3:**
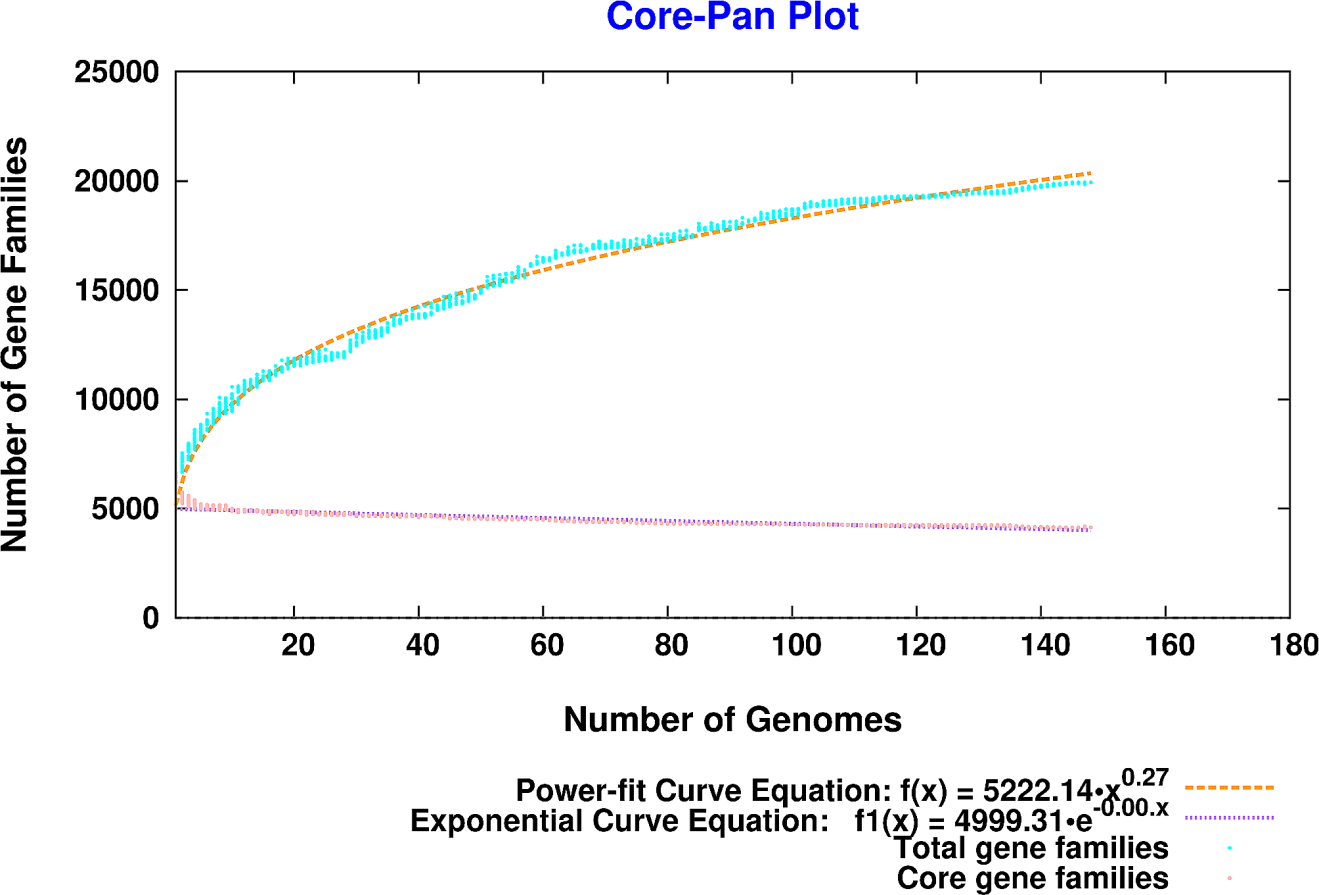
The pan and core genome plot of studied 148 *P. aeruginosa* genomes. Total gene families are shown by blue colour while pink colour represents core gene families

Protein sequences counted 953,346 from the 148 *P. aeruginosa* strains were examined for pangenome statistics. Figure 2 illustrates the distribution of coding DNA sequences concerning the genome size in all analysed sequenced strains with respect to countries. Among these, core CDSs were 4138, the 0.434% of the total CDSs in the 148 analysed *Pseudomonas* strains. Gene families (pangenome) and shared gene families (core genome) are plotted for all genomes added sequentially. Using power-fit curve equation: f(x) = 5222.14·x0.27 and exponential curve equation: f1(x) = 4999.31 e-0. 00.x, the parameter ‘b’ value was noted b = 0.272219, indicating that the pan-genome is still opened and the number of accessory and unique genes might be added to the genome (Fig. 3).

It was previously believed that bacteria, including the genus *Pseudomonas*, should have a permanently open pan-genome due to natural evolution and horizontal gene transfer. The strain 34Pae36 (accession ID: CP095770) reported from Colombia comprised the highest number of proteins containing CDSs (6994) comprising 2630 accessory genes and 226 unique genes, while strain MCF363 contained the least number of protein-coding sequences (*n* = 5648) comprising 1479 accessory genes and 31 unique genes. A total number of 5742 unique genes were identified in all strains. Of these, the greatest number of genes (*n* = 298) were noted in ZBX-P21 (accession ID: JACWGQ01), and the maximum number of genes (*n* = 96) were exclusively absent in CMC-115 (acc ID. CP046602) that is reported from the USA. In an in-house sequenced *P. aeruginosa* Emara01 strain, out of 5754 protein-coding sequences, 1601 genes were accessory, 15 were unique and, seven were missing. None of the genes were exclusively absent in 85 strains, the singleton gene was absent in 19 strains, and more than ten genes were missed in 15 strains (Table 2).

**Table 2:** Pangenome statistics of inhouse sequenced EMARA01 strain and 147 other reference strains

Strain	Accession ID	Country	No. of core genes	No. of accessory genes	No. of unique genes	No. of exclusively absent genes
VRFPA04	CP008739	India	4138	2128	90	19
PA99	CP042967	Thailand	4138	2183	149	54
PAO1	AE004091	USA	4138	1557	11	3
AG1	CP045739	Costa Rica	4138	2483	5	0
ST773	CP041945	USA	4138	2251	13	1
1811-13R031	CP046061	China	4138	2605	0	0
1811-18R001	CP046060	China	4138	2601	2	0
CMC-115	CP046602	USA	4138	1516	165	96
PAC6	CP053705	Taiwan	4138	1740	72	8
PAC1	CP053706	Taiwan	4138	2468	281	1
PSE6684	CP053917	South Korea	4138	2150	18	1
CDN118	CP054591	Nigeria	4138	2047	32	0
SE5458	CP046406	China	4138	2319	42	2
NCGM1984	AP014646	Japan	4138	1957	3	0
NCGM1900	AP014622	Japan	4138	1918	3	0
CMC-097	CP065848	USA	4138	2249	90	1
1.9E + 09	CP060392	China	4138	2088	63	0
152962	CP069198	France	4138	2216	19	0
B17932	CP070471	India	4138	2092	0	0
B17416	CP070467	India	4138	2088	4	0
PAS6	CP065947	China	4138	2028	14	0
PAM68	CP065948	China	4138	1913	7	12
ZBX-P23	CP061777	Lebanon	4138	2306	1	1
ZBX-P13	CP061778	Lebanon	4138	2126	21	0
ZBX-P12	CP061779	Lebanon	4138	2255	3	0
ZBX-P11	CP061780	Lebanon	4138	1982	48	0
PA790	CP075176	India	4138	2288	4	0
CCBH28525	CP086064	Brazil	4138	2264	118	0
TL3773	CP080011	China	4138	1919	83	55
P96131	CP087673	Sierra Leone	4138	2304	48	0
P4970C	CP087674	Sierra Leone	4138	2285	1	0
P93127	CP087675	Sierra Leone	4138	2229	12	1
PA1_NCHU	CP093395	Taiwan	4138	1840	69	1
PAD8	CP061073	China	4138	1893	46	0
34Pae36	CP095770	Colombia	4138	2630	226	0
34Pae8	CP095774	Colombia	4138	2094	51	0
NCGM257	AP014651	Japan	4138	2287	90	0
PA-1	CP097709	Chile	4138	2337	0	0
PA-2	CP097710	Chile	4138	2338	1	0
M27432	CP101885	Argentina	4138	2147	35	0
Pa3	CP086213	Belgium	4138	2392	88	0
2019CK-00034	CP107257	USA	4138	2271	107	18
MF1	CP117527	Chile	4138	2282	35	3
2022CK-00828	CP117749	USA	4138	2111	25	18
PASGNDM345	CP020703	Singapore	4138	2319	0	0
PASGNDM699	CP020704	Singapore	4138	2346	7	0
Pa58	CP021775	Mexico	4138	2488	87	2
Pa127	CP022000	Mexico	4138	2307	78	0
Pa1242	CP022002	Mexico	4138	2261	193	4
Pa1207	CP022001	Mexico	4138	2454	196	7
97	CP031449	Ghana	4138	2413	42	0
CCUG 70744	CP023255	Sweden	4138	2296	14	7
Y89	CP030913	South Korea	4138	2179	6	1
Y82	CP030912	South Korea	4138	2256	19	0
Y31	CP030910	South Korea	4138	1969	145	0
Y71	CP030911	South Korea	4138	2201	12	2
PABL048	CP039293	USA	4138	2654	97	0
PABL017	CP031660	USA	4138	1772	93	0
BA15561	CP033432	India	4138	2100	67	12
SP4528	CP033439	India	4138	2192	21	10
T2101	NSPN01	Thailand	4138	2470	61	17
IMP-13	CP034354	Belgium	4138	2357	78	4
SP4371	CP034369	India	4138	2262	19	10
SP2230	CP034434	India	4138	2238	68	9
GIMC5015:PAKB6	CP034429	Russia	4138	1579	6	14
SP4527	CP034409	India	4138	2265	74	4
paerg002	LR130527	Switzerland	4138	1762	15	0
paerg004	LR130531	Switzerland	4138	1755	7	0
paerg003	LR130530	Switzerland	4138	1763	1	0
paerg005	LR130534	Switzerland	4138	2154	5	0
paerg009	LR130533	Switzerland	4138	2142	16	9
paerg010	LR130536	Switzerland	4138	1766	0	0
paerg012	LR130537	Switzerland	4138	1756	0	1
CCBH4851	JPSS01	Brazil	4138	2005	31	38
AES1M	CP037925	Australia	4138	1743	3	0
AES1R	CP037926	Australia	4138	1754	20	2
60503	CP041774	China	4138	2131	23	2
A681	CP041771	China	4138	1967	46	0
243931	CP041772	China	4138	1833	23	5
CCUG 51971	CP043328	Sweden	4138	2260	13	0
ZBX-P25	JACWGN01	Lebanon	4138	2072	62	4
ZBX-P24	JACWGO01	Lebanon	4138	2162	23	0
ZBX-P22	JACWGP01	Lebanon	4138	2027	8	5
ZBX-P21	JACWGQ01	Lebanon	4138	1915	298	0
ZBX-P19	JACWGS01	Lebanon	4138	2110	1	0
ZBX-P16	JACWGV01	Lebanon	4138	2076	6	1
ZBX-P14	JACWGX01	Lebanon	4138	2185	50	0
ZBX-P10	JACWGY01	Lebanon	4138	2109	9	0
ZBX-P7	JACWHB01	Lebanon	4138	2089	2	1
ZBX-P1	JACWHH01	Lebanon	4138	2083	13	12
ps143	JAHVZI01	Saudi Arabia	4138	1632	147	1
ps147	JAHVZM01	Saudi Arabia	4138	1723	75	0
ps150	JAHVZP01	Saudi Arabia	4138	1571	32	0
ps153	JAHVZS01	Saudi Arabia	4138	1596	23	0
ps154	JAHVZT01	Saudi Arabia	4138	1751	19	1
ps159	JAHVZY01	Saudi Arabia	4138	2065	45	0
ps180	JAHWAM01	Saudi Arabia	4138	1637	12	0
Ps195	JAHWAZ01	Saudi Arabia	4138	1869	17	14
Ps202	JAHWBF01	Saudi Arabia	4138	1780	2	0
Ps203	JAHWBG01	Saudi Arabia	4138	1932	20	0
HARRAN_VET_OK28	JAKGDP01	Turkyia	4138	1730	296	1
ADU_VET_ST30	JAKGDR01	Turkyia	4138	1521	58	0
ADU_VET_ST3	JAKGDU01	Turkyia	4138	1973	26	0
P32	JAPWKK01	Egypt	4138	1780	3	1
P31	JAPWKL01	Egypt	4138	2247	11	0
P30	JAPWKM01	Egypt	4138	1997	0	0
P29	JAPWKN01	Egypt	4138	1894	38	0
P28	JAPWKO01	Egypt	4138	1565	25	3
P27	JAPWKP01	Egypt	4138	2259	8	1
P26	JAPWKQ01	Egypt	4138	1993	0	0
P25	JAPWKR01	Egypt	4138	1761	0	0
P24	JAPWKS01	Egypt	4138	1753	8	0
P23	JAPWKT01	Egypt	4138	1942	5	1
P22	JAPWKU01	Egypt	4138	2158	0	0
P20	JAPWKV01	Egypt	4138	1996	0	0
P19	JAPWKW01	Egypt	4138	1726	2	0
P18	JAPWKX01	Egypt	4138	1727	0	0
P17	JAPWKY01	Egypt	4138	1947	0	0
P16	JAPWKZ01	Egypt	4138	2083	18	0
P15	JAPWLA01	Egypt	4138	1733	9	0
P14	JAPWLB01	Egypt	4138	1995	0	0
P13	JAPWLC01	Egypt	4138	1619	29	5
P12	JAPWLD01	Egypt	4138	1730	5	2
P11	JAPWLE01	Egypt	4138	1809	2	0
P10	JAPWLF01	Egypt	4138	2589	85	0
P9	JAPWLG01	Egypt	4138	2222	6	2
P8	JAPWLH01	Egypt	4138	2167	39	0
P7	JAPWLI01	Egypt	4138	1748	1	0
P6	JAPWLJ01	Egypt	4138	2157	0	0
P5	JAPWLK01	Egypt	4138	2017	14	0
P4	JAPWLL01	Egypt	4138	1726	1	0
P3	JAPWLM01	Egypt	4138	2156	0	0
P2	JAPWLN01	Egypt	4138	2528	22	5
P1	JAPWLO01	Egypt	4138	2154	1	0
AZPAE14697	JTXI01	Israel	4138	1615	88	45
AZPAE14695	JTXJ01	Israel	4138	1833	59	26
WH-SGI-V-07276	LLUC01	Turkyia	4138	1851	9	0
WH-SGI-V-07277	LLUD01	Turkyia	4138	1864	54	1
WH-SGI-V-07278	LLUE01	Turkyia	4138	1898	36	1
Kasamber	MVDK01	Sudan	4138	1790	43	6
MCF363	NSMU01	Israel	4138	1479	31	0
Emara01	JARQZF01	Inhouse Egypt	4138	1601	15	7
GCID_CRE_0006	RYXU01	Egypt	4138	1959	23	4
NUBRI-P	SMZF01	Sudan	4138	1656	26	2
KOL14.W.495.36	WIFR01	Greece	4138	1636	0	0
KOL14.W.495.35	WIFS01	Greece	4138	1636	0	0
KOL14.W.495.34	WIFV01	Greece	4138	1643	0	0
KOL14.W.495.33	WIGC01	Greece	4138	1637	1	0

The phylogenetic relationships between *P. aeruginosa* EMARA01 and a total of 147 additional *P. aeruginosa* reference strains were examined to identify the genomic epidemiological features of *P. aeruginosa* EMARA01 in the context of the world. *P. aeruginosa* has a dynamic genetic makeup that enables it to colonize a wide range of environments, including humans, where it can cause opportunistic infections [66].

**Fig. 4:**
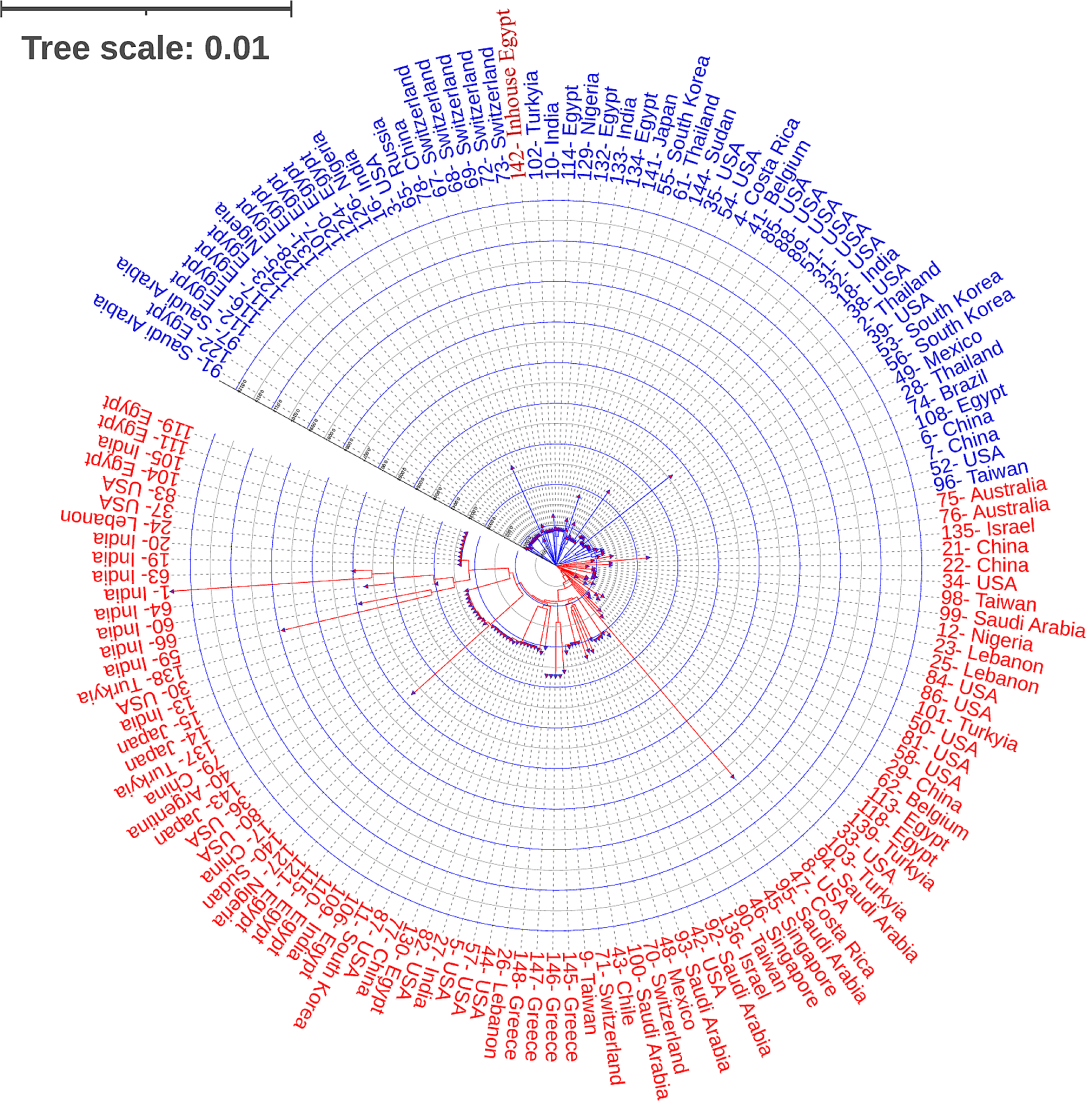
Core genome MLST phylogenetic analysis of the 148 *P. aeruginosa* strains

The genomic characteristics of *P. aeruginosa* EMARA01 are supported by the randomly selected reference strains reported from other regions [67, 68]. Compared to one of the most popular reference strains, *P. aeruginosa* PAO1, the *P. aeruginosa* EMARA01 exhibits 99.51% average nucleotide identity. EMARA01 strain genome exhibits a close evolutionary relationship with many other *P. aeruginosa* strains isolated from various clinical samples from countries like Mexico, Sweden, Egypt, Senegal, Brazil, and Saudi Arabia (Fig. 4). The phylogenetic analysis revealed that EMARA01 exhibits the closest relationship with the CCBH28525 strain isolated from the tracheal secretion of patients admitted to the hospital in Bahia state Brazil [69, 70].

### Biological function and Pathways Identification

**Fig. 5:**
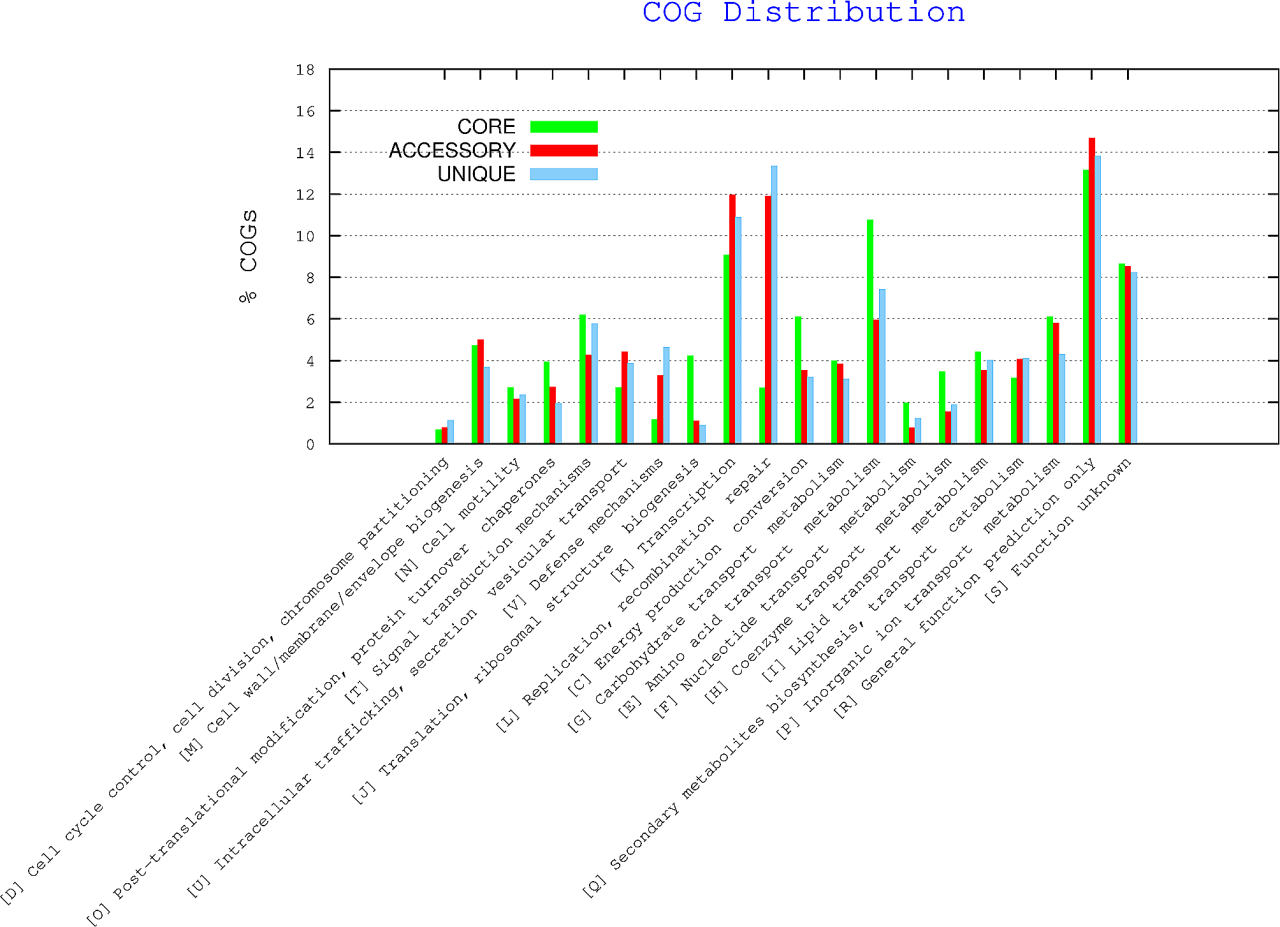
Cluster of orthologous groups distribution of the genes making up core, accessory and unique portion of the studied genomes

The COG analysis of the pan-genome revealed that the highest number of genes in the core genome belongs to the prediction of general function, followed by amino acid transport metabolism and transcription (Fig. 5). Similar findings from earlier research have been found for *P. aeruginosa* core and accessory genomes, with the core genome being enriched in central metabolism functions and important cellular processes like replication, transcription, and translation as well as other related biosynthetic pathways [71, 72].

RAST-based annotation identified 393 subsystems for functional genes and metabolic pathway connections in the EMARA01 strain. Most of the genes were involved in amino acid metabolism (501 genes), followed by genes involved in carbohydrates (*n* = 288), protein metabolism (*n* = 220 genes), the synthesis of cofactors, vitamins, prosthetic groups, and pigments (*n* = 200), several genes were involved in membrane transport, fatty acids, lipids, and isoprenoids, aromatic compounds, response to stress and respiration. According to the subsystem analysis, 62 genes are associated with disease, virulence, and defence. Of these, were responsible for toxic and antibiotic resistance, 17 were involved in invasion and intracellular resistance, and six were associated with the antibacterial peptides. We confirmed multiple earlier investigations by identifying distinct genes and proteins from the whole genome of *P. aeruginosa* EMARA01 that are attributed to various subsystem metabolic activities [73, 74]. Our study presents a significant increase in metabolic functional genes and the pathways crucial to the emergence of *P. aeruginosa* infection.

## Conclusions

This study provides valuable information on *P. aeruginosa* isolates obtained from liver transplant patients and their antibiotic resistance profiles. The genomic characterization and pan-genome analysis contribute to our understanding of the genetic diversity and functional aspects of *P. aeruginosa* genes. These findings may have implications for infection control measures and the development of targeted therapies in the context of liver transplant patients. Future research and surveillance efforts should focus on monitoring the prevalence and dissemination of extensively drug-resistant *P. aeruginosa* strains, and novel therapeutic approaches targeting the identified AMR genes and pathways should be explored. Ultimately, this knowledge will contribute to improved patient outcomes and the prevention of infections in this vulnerable population.

### Electronic supplementary material

Below is the link to the electronic supplementary material.

Supplementary Material 1

Supplementary Material 2

## Data Availability

Sequencing data are available at the Sequence Read Archive (NCBI) the 16 S rRNA sequence is deposited under accession number OQ976904 and the assembled genome of *P. aeruginosa* EMARA01 is uploaded under the accession identifier JARQZF000000000.1.

## Abbreviations

AMR: Antimicrobial Resistance
AST: Antibiotic susceptibility testing
BPGA: Bacterial Pan Genome Analysis
COG: Cluster of Orthologous Genes
CLED: Cystine lactose electrolyte deficient agar
CLSI: Clinical and Laboratory Standards Institute
ESLD: End-stage liver disorder
GEC: Gastroenterology Surgical Center
KEGG: Kyoto Encyclopaedia of Genes and Genomes
MDR: Multi-drug resistant
PGAP: Prokaryotic Genome Annotation Pipeline
UTIs: Urinary tract infections
WGS: Whole genome sequencing
XDR: Extensively drug-resistant
