# Supplementary material for: Microbiological and molecular studies on a multidrug-resistant Pseudomonas aeruginosa from a liver transplant patient with urinary tract infection in Egypt
Source: BMC Microbiol. 2024 May 27;24:184. doi: 10.1186/s12866-024-03318-0 (PMC11129433; doi:10.1186/s12866-024-03318-0)
Supplement: Supplementary file 1 — Supplementary Material 1 [file 12866_2024_3318_MOESM1_ESM.docx]

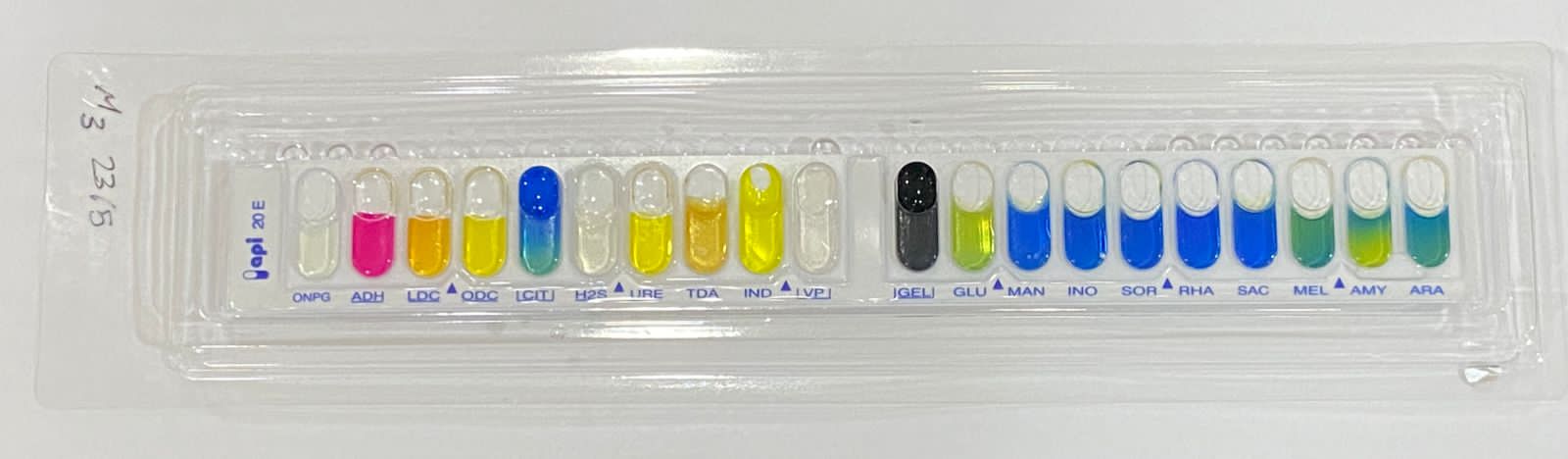


**Figure S1.** Biochemical identification of *P. aeruginosa* EMARA01 using API.


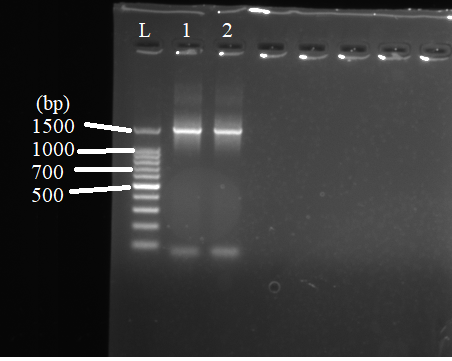


**Figure S2.** Amplification of targeted DNA using universal primers.


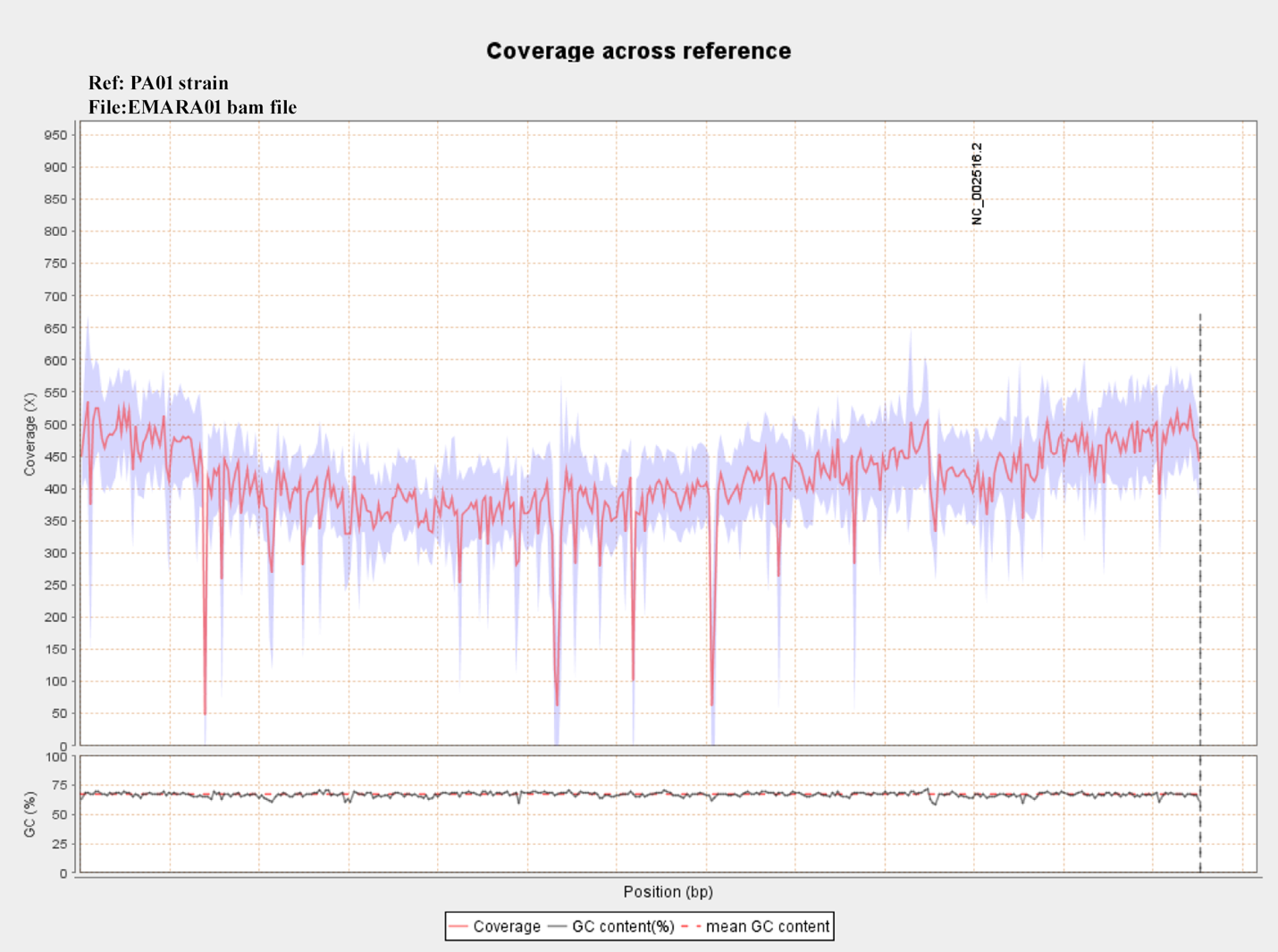


**Figure S3.** Genome coverage of sequenced EMARA01 strain against the reference genome PAO1 strain.
